# Rupture of the Stomach in a Boy

**Published:** 1846-06

**Authors:** J. Stewart Allen

**Affiliations:** Resident Surgeon to the St. Marylebone Infirmary


*Rupture of the Stomach in a Boy.* By J. Stewart Allen, Esq., Resident Surgeon to the St. Marylebone Infirmary. On Thursday, December 11th, 1845, an inquest was held at the St. Marylebone Infirmary, before Mr. Wakley, M.P., on the body of a boy named James Geogehan, aged 10 years.

The boy had been an inmate of the workhouse for upwards of eight years, during which time he had been frequently ailing, but on the Sunday previous to his death he had appeared in his usual health. On Sunday night, about 12 o'clock, the nurse of the school heard him moaning in bed, and afterwards going to the water-closet. She rose and questioned him. He complained of extreme pain over the abdomen, which was much enlarged. He was brought over to the Infirmary about a quarter before 1 o'clock on Monday morning. When admitted, he was in a state of collapse, countenance sunk, extremities cold, and pulse not perceptible at the wrist; he had an increasing desire to vomit, but could bring nothing up; the abdomen was greatly distended, and very tense. Warmth was applied over the body, mustard sinapisms, turpentine fomentations, &c., and stimulants were administered internally, but without effect; he gradually sank, and died at half past 3 on the same morning, about four hours (as well as could be ascertained) after his first complaining of pain.

The following were the post-mortem appearances:

The abdomen was enormously distended, the false ribs being pushed outwards, quite altering the natural shape of the body; the lungs were healthy; the pericardium so intimately adhering to the heart that it could not be separated. On opening the abdomen, a rush of fœtid air took place; the stomach was found collapsed, much enlarged, and stretching across the abdomen; the small intestines were also much distended with air and semi-digested food. On raising the stomach, a rupture was found to have taken place at its cardiac extremity, through which a portion of the contents had escaped into the abdomen; the stomach still contained about a pint of pulpy semi-digested food. On removing the contents the mucous membrane was found to be soft, and presented a mottled appearance; the pyloric extremity was much thickened, and firmly contracted, inclosing in its folds two small pieces of potato; so close was the contraction, that when the stomach was filled with water, it would not permit a drop to pass. The cæcum was contracted, and throughout, the calibre of the large intestines was very small, in some places not being wider than a common quill; there were portions of the large intestines, each about an inch and a half in length, of a dull reddish color; there was considerable congestion of the veins on the surface of the brain, but in other respects that organ was healthy.

This case is remarkable as one of spontaneous rupture without ulceration; but it should be remarked that the boy had for some years suffered from deficient nutrition; he generally ate largely, but seemed to derive little benefit from his food. The stomach has since been examined with the microscope. The white spots which gave the mucous membrane a mottled appearance, were found to be mucous follicles much enlarged, and distended with their natural secretion; the mucous membrane was soft, and loaded with fluid. These were the only morbid appearances that could be detected in the coats of the stomach.—*Ibid.*

